# Expert opinion as 'validation' of risk assessment applied to calf welfare

**DOI:** 10.1186/1751-0147-50-29

**Published:** 2008-07-14

**Authors:** Marc BM Bracke, Sandra A Edwards, Bas Engel, Willem G Buist, Bo Algers

**Affiliations:** 1 Animal Sciences Group, Wageningen University and Research Centre, P.O. Box 65, 8200 AB Lelystad, The Netherlands; 2 University of Newcastle, School of Agriculture, Food and Rural Development, King George VI Building, Newcastle upon Tyne, NE1 7RU, UK; 3 Department of Animal Environment and Health, Faculty of Veterinary Medicine, Swedish University of Agricultural Sciences, P.O. Box 234, SE-53223 Skara, Sweden

## Abstract

**Background:**

Recently, a Risk Assessment methodology was applied to animal welfare issues in a report of the European Food Safety Authority (EFSA) on intensively housed calves.

**Methods:**

Because this is a new and potentially influential approach to derive conclusions on animal welfare issues, a so-called semantic-modelling type 'validation' study was conducted by asking expert scientists, who had been involved or quoted in the report, to give welfare scores for housing systems and for welfare hazards.

**Results:**

Kendall's coefficient of concordance among experts (n = 24) was highly significant (P < 0.001), but low (0.29 and 0.18 for housing systems and hazards respectively). Overall correlations with EFSA scores were significant only for experts with a veterinary or mixed (veterinary and applied ethological) background. Significant differences in welfare scores were found between housing systems, between hazards, and between experts with different backgrounds. For example, veterinarians gave higher overall welfare scores for housing systems than ethologists did, probably reflecting a difference in their perception of animal welfare.

Systems with the lowest scores were veal calves kept individually in so-called "baby boxes" (veal crates) or in small groups, and feedlots. A suckler herd on pasture was rated as the best for calf welfare. The main hazards were related to underfeeding, inadequate colostrum intake, poor stockperson education, insufficient space, inadequate roughage, iron deficiency, inadequate ventilation, poor floor conditions and no bedding. Points for improvement of the Risk Assessment applied to animal welfare include linking information, reporting uncertainty and transparency about underlying values.

**Conclusion:**

The study provides novel information on expert opinion in relation to calf welfare and shows that Risk Assessment applied to animal welfare can benefit from a semantic modelling approach.

## Background

For several decades Risk Assessment has been conducted in the field of human and animal health [e.g. [1-5]]. The need to develop a formal means for Risk Analysis of animal welfare has been recognized at the European level [6,7]. A recently published report on qualitative Risk Assessment on intensively farmed calves [8,9] was an important step toward transparent decision making on animal welfare. The methodology, however, was also recognized to be in need of further modification [[2,8], p.8].

In a separate paper [10] we reported on a critical analysis from a semantic-modelling perspective, and formulated recommendations for improvement of Risk Assessment applied to animal welfare, as presented in the EFSA report [8,9]. Semantic modelling is a kind of risk-benefit assessment, i.e. welfare assessment based on a structured analysis of available scientific information [11-14]. Several semantic models have successfully been 'validated' against expert opinion [15-17]. In these studies, typically two sets of scores have been requested from experts: welfare scores for housing systems and scores for the importance of welfare-relevant system attributes, which we suggested being the equivalents of the 'hazards' in Risk Assessment [10]. Given their value in relation to semantic modelling, these expert-opinion scores probably provide a good starting point for representing expert reasoning about animal welfare. Conceptually, these sets of scores provide the first two steps in backward expert-reasoning from overall scores to the underlying scientific information: welfare scores for housing systems can, in principle, be explained by the attribute (i.e. hazard or risk) scores, which can be explained by the underlying science specifying relationships between two types of attributes, namely design criteria and welfare performance criteria [11].

In order to check a number of critical points raised in the main study such as the need to specify definitions, the need to include positive (behavioural) aspects of welfare and to complement an assessment of risk components with a perception of overall welfare [10], this paper reports on a study comparing the scores for Hazard Characterization (HC), Exposure Assessment (EA) and Risk Characterization (RC) as presented in the EFSA report with semantic-modelling type scores elicited from experts about a selected number of welfare hazards and housing systems for calves. This paper also addresses several additionally suggested points for improvement of Risk Analysis [10], including the linking of information (such as between hazards and underlying scientific information, and between HC scores and overall welfare scores), reporting of uncertainty measures, verification of items possibly lacking from EFSA [8,9] and transparency about underlying values. Finally, the welfare scores given by the experts, which had all been involved or cited in the EFSA report [8,9], provide complementary information to decision makers on the welfare of calves, and also provides unique information on how groups of experts may differ in assessing animal welfare.

The objectives of this paper, therefore, were to elicit expert opinion about calf welfare as part of a semantic modelling-type 'validation' study addressing the above-mentioned aspects of the Risk Assessment (RA) approach developed in the calf EFSA report [8,9].

## Methods

A survey was conducted in November-December 2006 by sending an email message to the authors of the EFSA report, to the veterinary experts who had given advice on Exposure Assessment (EA) and to a selected number of applied ethologists, who were the authors of papers cited in EFSA [9] (together representing three different roles in the EFSA report). In total 38 experts from 10 different (European and North-American) countries were contacted with the request to assess overall animal welfare of 11 housing systems (on a scale from 0, worst to 10, best) and 18 hazards (also on a scale from 0 to 10, i.e. least to most important for welfare). In the questionnaire, it was emphasized that only welfare was to be assessed, and that welfare could be defined as what matters to the animals from their point of view. The items were presented in a table-format (comparable to Tables 2 and 3 below, but providing the full description given in the EFSA report) in a randomized order. In addition, experts were asked to state their professional background and an opinion on the EFSA report [8,9]. Experts were then classified into those with a background in veterinary science, ethology, or of mixed background, i.e. with a background in both veterinary medicine and ethology. Item descriptions were identical to the ones used in the EFSA report [8,9], except for two newly added items in each list. White veal in baby boxes and suckler calves at pasture were added as 'controls' to the list of housing systems, and insufficient roughage and insufficient play were added to the list of hazards in order to examine the hypothesis that these are important systems and hazards not adequately addressed in the EFSA report [8,9] as indicated in Bracke et al. [10].

**Table 1:** Overview of abbreviations used

Abbreviation	Meaning
EA	Exposure assessment
EFSA	European Food Safety Authority
HC	Hazard characterisation
RC	Risk characterisation
SM	Semantic modelling

**Table 2:** Specification of numbers of respondents according to their background and their role in the writing of the EFSA (2006b) report.

	Background			
Role in EFSA report	Vet	Ethol.	Mixed	*Total*

Author of EFSA report	0	0	3	3
Veterinarian involved in EA	5	0	0	5
Contacted expert	3	0	1	4
Cited reference author	0	11	1	12

*Total*	8	11	5	24

Vet: veterinarian; Ethol.: Applied ethologist; Mixed: background both as Vet and as applied ethologist.

**Table 3:** Agreement among experts (expressed as W, Kendall's coefficients of concordance, for welfare scores given to the 11 housing systems and to the 18 hazards in the questionnaire), and agreement between experts and EFSA report (expressed as Rho, Spearman's rank correlation coefficients, between median expert scores and hazard/risk characterisation)

	Housing systems			Hazards			Hazards		Housing systems			
	
Type of expert							HC		Median RC		Total RC	
	W	P	n	W	P	n	Rho	P	Rho	P	Rho	P
	
All experts	0.29	0.00	18	0.18	0.00	21	0.47	0.06	-0.54	ns	-0.45	ns
Ethologists	0.23	0.01	9	0.11	0.04	9	0.28	ns	-0.03	ns	-0.22	ns
Veterinarians	0.10	ns	5	0.09	ns	7	0.57	0.02	-0.68	0.05	-0.36	ns
Mixed background	0.15	ns	4	0.34	0.00	5	0.66	0.01	0.13	ns	-0.08	ns

P: significance level; ns: not significant; n: number of experts without missing values; HC: Hazard Characterisation; RC: Risk Characterization.

Kendall's coefficient of concordance was calculated to determine agreement among experts, and Spearman's correlation coefficients (Rho) were used to determine relationships between median expert scores and Hazard Characterization (HC) scores for hazards, and between expert scores and overall Risk Characterization (RC) scores for housing systems. Hazard scores from the survey were compared with HC scores, because these are indicators of the potential importance of a hazard. In the EFSA report, HC scores were constant across housing systems. RC scores were calculated in the EFSA report by multiplying HC and EA (Exposures Assessment) scores (see Table 1 for an overview of abbreviations used). RC scores indicate various levels of hazard exposure and risk related to hazards in different housing systems. Overall risk per housing system was calculated from the median and total (i.e. the sum of components) RC scores reported for each housing system in EFSA [8,9]. These sets of scores differed because not all hazards were scored for all systems. Both scores only give a rough idea of the overall risk, as the underlying scales were not cardinal (i.e. the interval between successive points of the scale may not have been constant).

The statistical analyses were done in SPSS 13.0 [18]. Correlations for housing systems were expected to be negative, because higher expert scores implied higher welfare, whereas higher RC scores implied more risk for welfare, i.e. lower welfare.

To determine main factor effects on the scores given by the experts, a components of variance model was used [19], initially ignoring the fact that scores ranged from 0 to 10. The model comprised random effects for experts and fixed effects for Hazard/Housing system, Role and Background as main effects. The additional factor Gender (of the expert) and two-factor interactions were systematically tested, dropping additional factor combinations when not significant. The most relevant models were subsequently analyzed with a threshold model comprising the aforementioned fixed and random effects. The estimation procedure is discussed in Keen and Engel [20] where it is shown that this model is appropriate for analyzing ordered scores. In the analyses, the following factors were considered: Housing system (n = 11) or Hazard (n = 18); Background (veterinarian, n = 8; applied ethologist, which often combined the study of animal behaviour and animal science, n = 11; and mixed background, which were mostly veterinarians working as applied ethologist, n = 5); Gender (male, n = 16; female, n = 8); Role (i.e. role of involvement in the writing of the EFSA report [9]; these included Working Group member, i.e. authors of the report, n = 3; veterinarian contributing to Exposure Assessment, n = 5; other contacted expert, e.g. by being acknowledged in the report, n = 4; and author of a reference quoted in EFSA [9], n = 12 for housing systems and n = 11 for hazards). The interaction between Role and Background could not be examined because they were confounded, e.g. reference authors were all ethologists and exposure assessors were all veterinarians (see Table 2).

Significance levels were determined with Wald tests employing a chi-square approximation [21]. Calculations were performed with GenStat [22].

## Results

The response rate of the questionnaire was 63% (n = 24 respondents for housing systems and n = 23 for hazards), comprising 3 Working Group members, 5 exposure assessors and 16 other scientific experts. In total, ten experts were positive about the Risk Assessment approach in the EFSA report. The other experts either did not respond to this question or stated that they were not familiar with the report. Working Group members (i.e. authors of the report) and exposure assessors generally responded positively, whilst 70% of respondents not personally involved (i.e. only through having a reference cited in the EFSA report) indicated that they were not familiar with the report (that had only recently been published at the time the survey was conducted). Several experts expressed doubt about the scientific value of the questionnaire (e.g. for requesting an instantaneous response without prolonged contemplation). Several experts complained about the vague descriptions of the housing systems, and some experts perceived hazards to be non-uniform (e.g. castration versus floors).

**Figure 1:**
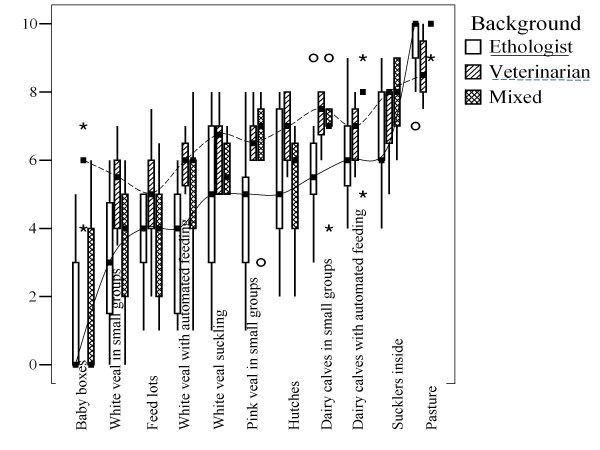
**Boxplot of welfare scores for housing systems by background (see also Table 2, n = 24 experts)**. Asterisks and circles indicate two types of outliers identified as standard practice in SPSS. Outliers are scores with values between 1.5 and 3 box lengths from the upper or lower edge of the box. The box length is the interquartile range (i.e. median 25% to 75% of values), while the horizontal line in the box indicates the median value. The two curved lines are connecting median values of ethologists (solid line) and veterinarians (dashed line) respectively.

**Figure 2:**
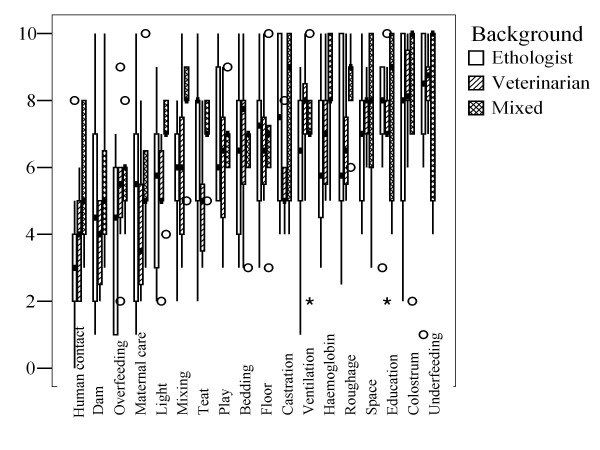
**Boxplot of scores for hazard importance by background (see also Table 3; n = 23 experts)**. Asterisks and circles indicate two types of outliers as standard practice in SPSS. Outliers are scores with values between 1.5 and 3 box lengths from the upper or lower edge of the box. The box length is the interquartile range (i.e. median 25% to 75% of values), while the horizontal line in the box indicates the median value.

Figures 1 and 2 give boxplots of the housing and hazard scores given by the experts, grouped by their professional background. Figure 1, for example, shows that median welfare scores for the housing system 'baby boxes' were 0.0, 6.0 and 0.0 for ethologists, veterinarians and experts with a mixed (veterinary and ethological) background respectively.

Table 3 shows Kendall's coefficient of concordance (W) for the scores given to housing systems and hazards by background. When considered together, there was low (W = 0.29; W = 0.18), but highly significant (P < 0.001) agreement among the whole group of experts. Agreement was generally less significant when examined within the smaller subgroups of experts with different backgrounds. Table 3 also shows Spearman's correlation coefficients (Rho) between (group and subgroup) expert opinion scores and EFSA scores (i.e. HC scores for hazards and RC scores for housing systems respectively). Significant correlations were found only for HC scores (reported in EFSA) and (the hazard scores given by) veterinarians (Rho = 0.57), for HC scores and mixed-background experts (Rho = 0.66), and for median RC scores and (the housing system scores given by) veterinarians (Rho = -0.68).

**Figure 3:**
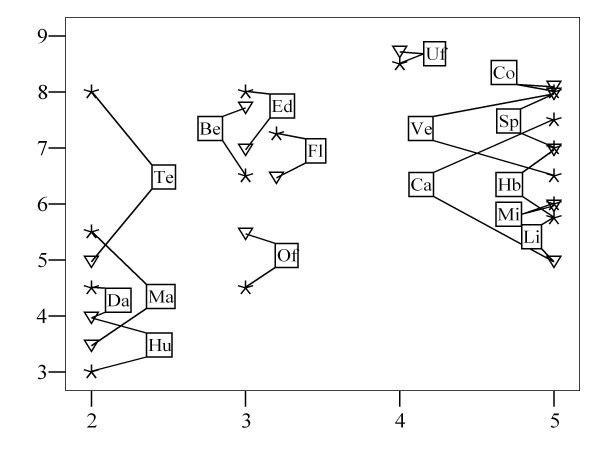
**Scatter plot of HC scores (horizontal axis) and median hazard scores (y-axis) given by veterinarians (triangles) and ethologists (stars)**. Hazard codes: Be: Bedding; Ca: Castration; Co: Colostrum; Da: Dam; Ed: Education; Fl: Floor; Hb: Haemoglobin; Hu: Human contact; Li: Light; Ma: Maternal care; Mi: Mixing; Of: Overfeeding; Pl: Play; Ro: Roughage; Sp: Space; Te: Teat; Uf: Underfeeding; Ve: Ventilation (see also Table 5).

Figure 3 illustrates two relationships found for HC and hazard scores for experts with different backgrounds, namely ethologists (where the relationship was not significant) and veterinarians (where Rho was significant, namely 0.57, see Table 3). Figure 3 shows hazards that received a high HC score in EFSA (2006a, b), but received relatively low expert scores (for both veterinarians and ethologists), such as light (Li) and mixing of calves (Mi). It also illustrates the reverse, especially for access to a natural teat (Te) (particularly for ethologists) and for education (Ed), bedding (Be) and floor (Fl) (both types of expert).

In the components of variance models, effects of Gender (main effects and interactions) were neither significant for housing-system scores nor for hazard scores. For hazard scores, no significant interactions were found, resulting in a model with main effects for Hazard (P = 0.00), Role (P = 0.33) and Background (P = 0.08). For housing-system scores, the final model comprised Housing system (P < 0.05), Role (P = 0.01), Background (P < 0.05) and the interaction between Housing system and Background (P < 0.01).

In the final threshold model for hazard scores, only Hazard was significant (P < 0.001; see Table 5). Role was not significant, and a trend was found for Background (P = 0.06). Respondents with a mixed background tended to give higher hazard scores than veterinarians, and ethologists gave intermediate scores that were closer to the mixed-background group.

According to the experts, the least important hazards were insufficient human contact, separation from the dam, overfeeding and lack of maternal care (Table 5). These did not significantly differ from each other, and scored significantly lower than all other hazards, except for light which was intermediate. A whole range of hazards with somewhat higher scores did not significantly differ from each other. The 6 most important hazards were underfeeding, inadequate colostrum intake, poor education, insufficient space, inadequate roughage and iron deficiency (in that order, see Table 5). In this list, underfeeding scored significantly higher than iron deficiency. The two hazards added for 'validation' purposes, insufficient space to play and inadequate roughage, ended up in the middle and middle-upper range respectively.

In the final threshold model for Housing-system scores, the interaction between Housing system and Background failed to reach significance. This left a model with only main effects for Housing system (P < 0.001; see Table 4), Role (P = 0.03) and Background (P = 0.03).

**Table 4:** Descriptions of housing systems, their median scores and significance levels (Sig.) according to the final threshold model (see text).

Description of housing systems	Median	Sig.
White veal housed individually in baby boxes (first 6–8 weeks), bucket fed (ie not suckling)	3.00	a
White veal in small groups, bucket fed (ie not suckling)	4.00	bc
Feed lots (high density groups within outside pen)	4.00	c
White veal in larger groups, with automatic feeding (ie not suckling)	5.00	ce
White veal in small groups, suckling	5.50	deg
Pink veal in small groups, bucket fed + (some) solid fods, not suckling	6.00	fg
Hutches outside with replacement dairy calves, bucket fed (not suckling) + solid foods, weaned at 2–3 months	6.00	gi
Small groups of replacement dairy calves, bucket fed (not suckling) + solid foods, weaned at 2–3 months	7.00	hik
Groups of dairy calves with an automatic feeding system (not suckling) + solid foods, weaned at 2–3 months	7.00	ik
Suckler beef calves in groups kept inside, led twice a day to the dam for suckling up to 6–9 months	7.00	jk
Suckler beef calves kept with cows in a herd at pasture	9.50	l

For significance levels (Sig.), systems without a common letter differ significantly (P < 0.05).

**Table 5:** Descriptions of hazards, their median scores and significance levels (Sig.) according to the final threshold model (see text).

Description of hazards	Median	Sig.
Insufficient contact with humans	4.00	a
Separation from the dam	4.00	ac
Too rich diet (overfeeding)	5.00	ac
Lack of maternal care	5.00	ac
Insufficient light	6.00	bce
Mixing calves from different sources	6.00	deg
No access to natural or artificial teat	6.50	eg
Insufficient space for natural play (eg running and gamboling)	6.00	eg
No bedding	7.00	dg
Poor floor condition	7.00	fg
Castration/dehorning, no anaesthetics	6.00	fi
Inadequate/inappropriate ventilation	7.00	fi
Iron deficiency	7.00	fi
Insufficient/inadequate roughage	7.00	gik
Insufficient floor space allowance	7.50	gik
Poorly educated stockperson	7.50	gik
Inadequate colostrum intake	8.25	hik
Underfeeding	9.00	jk

For significance levels, systems without a common letter differ significantly (P < 0.05).

The various veal and feedlot systems received the lowest scores, with (white veal calves in) Baby boxes (the system added for 'validation' purposes as a negative control) scoring significantly lower than the other systems (see Table 4). Pink veal and white veal suckling from a dam scored significantly higher than similar bucket-fed groups of white veal calves. Suckler beef calves kept on pasture (the system added for 'validation purposes as a positive control) scored significantly higher than all other systems.

Veterinarians gave significantly higher overall welfare scores for housing systems compared with mixed-background experts and ethologists, but the latter did not differ from each other.

Working Group members (i.e. authors of the EFSA report) did not significantly differ from reference authors, but Working Group members did give significantly higher overall welfare scores than veterinary exposure assessors and contacted experts.

## Discussion

The objectives of this paper were to elicit expert opinion about calf welfare and to verify conclusions from our previous analysis [10] of the new Risk Assessment (RA) approach developed in the calf-welfare report of the European Food Safety Authority [8,9]. This paper reports a first validation-type study of Risk Assessment applied to animal welfare, which is a methodology in need of further refinement [[8], p.8; [2]] to which end recommendations from a semantic-modelling perspective have been formulated [10].

### Methods

A semantic-modelling type questionnaire [15-17] was sent to experts, requesting 'intuitive' welfare scores for housing systems and hazards on scales from 0 to 10. The total number of experts was limited. The experts in this study were all applied ethologists or veterinary scientists that had been involved or cited in the EFSA [9] report on the welfare of intensively-reared calves. These scientists had been identified in the EFSA report as *the *experts on this subject in Europe. From a semantic modelling (SM) perspective, however, the term 'experts' must be qualified, because the respondents all had a particular area of expertise (rather than being complete and fully impartial generalists), and few experts had experience with (the technical details of) (semi-)quantified overall welfare assessment as developed, for example, in SM. This may limit the extent to which the survey can be regarded as a 'gold standard'. In the section 'hazards' below this point will be further illustrated with the example of 'underfeeding'.

In response to the questionnaire, several experts questioned its scientific value. Perhaps these respondents had not fully realized that in this study *they *were the experimental subjects. By virtue of being knowledgeable experts, their opinion, even when elicited in this stimulus-response like fashion, was inherently valid (by being their expert opinion), especially also because uncertainties about the scores were to become part of the (biological) variation around the group's opinions. These respondents' complaint, however, indicates a legitimate concern about a risk of misinterpretation of the outcomes of this study. When experts believe that one item, housing system or hazard, is better or more important than another, it does not logically follow that it actually is better or more important. For the latter conclusion, further scientific studies are needed, esp. including measurements of animal-based attributes. SM subscribes to that view, but also recognizes that an assessment of animal welfare is always an assessment from a human's point of view [23]. It is rarely possible to assess overall welfare within a single scientific study, and it always requires taking a range of (animal- and environment-based) measures that must be selected and interpreted within the context of decades of scientific research. As far as we know, the most structured way available at present to move towards that objective is semantic modelling.

Furthermore, the respondents validly complained about the vague and general descriptions provided for hazards and housing systems. Unfortunately, these were unavoidable in this study because they had to be adopted from the EFSA report [8,9] that was under scrutiny. As indicated in the underlying study [10] also from a SM perspective, more detailed descriptions would be required: hazards should be specified in relation to the underlying scientific information and housing systems should be specified using a matrix of welfare-relevant attributes covering the range of conditions prevailing in the housing systems in the assessment domain, including both environment-based inputs and animal-based outcomes covering all welfare-relevant needs [13,15].

A further methodological issue concerns the concept of Risk. Risk may differ from welfare assessment in that a risk to welfare may or may not actually compromise welfare, depending on the (negative welfare) effects actually occurring. However, because both survey and EFSA report [8,9] concerned the European scale, the population of farms was sufficiently large to assume that risks and their associated effects on welfare were (more or less) referring to the same properties of the system. Exactly which properties the respondents considered cannot be determined from this survey. As the respondents were familiar with the housing systems and hazards (as they were experts who had been asked to abstain from scoring when they were not familiar with it) and as they were asked to respond without much contemplation, it is reasonable to assume that in most cases the scores were given for typical, representative examples of systems and hazards.

An important caveat with respect to the interpretation of housing-system (and hazard) scores, however, is that the scores were given for the experts' personal interpretation of welfare. Even though welfare was defined in the survey as what matters to the animals from their point of view, differences in interpretation may have contributed to variation in the scores. By contrast, whereas the scores were probably given for 'average' systems, this survey did not address the potentially much larger range of variation existing between individual farms within type of system. In relation to this variation, one expert commented that 'a good farmer can produce good welfare in a poor system'. Though this statement can be challenged, the reverse is certainly true: a bad farmer will cause poor welfare in what is otherwise a good system. Therefore, the scores reported here for the different types of housing systems and hazards cannot be taken to represent welfare scores for all individual cases, and further work is needed to address this point.

Finally, welfare scores for housing systems and hazards were expressed on a scale from 0 to 10. A median score of 5 was previously found to be the cut-off point for acceptability proposed by experts who had given welfare scores for enrichment materials for pigs [16]. This supports a tentative suggestion to use some score in the middle of the scale (somewhere around 5) as the (implicit) cut-off point for what the experts in the present survey may have considered acceptable/important, also because this would be in accordance with its familiar use as a grading scale, e.g. in schools and psychological tests.

### General 'validation'

Kendall's coefficients of concordance (W) were highest (0.34, P < 0.001) for hazard scores given by mixed-background experts, which is explained by the fact that these were experts that had been involved as authors of the EFSA report [9]. Otherwise, W values were low for both housing system and hazard scores (range 0.09 to 0.29, Table 3) compared to similar welfare scores for pregnant sows (W = 0.73 and 0.43 for housing systems and attributes respectively, [15]). Nevertheless, W values were highly significant for the whole group of experts, probably due to the larger number of individuals in the dataset. Vague item descriptions and the request to provide intuitive scores may have contributed to this finding. More contemplation about better specified items, e.g. in working group discussions, may improve the level of concordance (but see [10]), and this could be monitored with a semantic-modelling type questionnaire. As long as the objective of complete consensus has not been reached, the level of concordance among the experts and the degree of variation in the scores given may provide an entry for specifying the level of uncertainty for scores given in Risk Assessment.

Compared to previous studies validating semantic models against expert opinion [15-17], this study yielded moderate correlations for hazards (range 0.28–0.66) and relatively poor and many not significant correlations for housing systems. The correlation for HC scores was highest for experts with a mixed background, followed by veterinarians (Rho: 0.66 and 0.57, both P < 0.05). Surprisingly, the median scores for hazard importance provided by ethologists did not correlate significantly with the HC scores reported in EFSA [8,9]. This may be explained by the confounding relationship with Role: many ethologists had not directly been involved in the writing of the report (they had only been cited), whereas veterinarians and mixed-background experts in this study had been actively involved as exposure assessors and as authors of the report respectively.

Poor correlations between expert opinion and Risk Assessment may reflect the latter's focus on negative hazards, rather than on both negative and positive welfare aspects, and it may reflect the RA's focus on component hazards rather than on overall (risk for poor) welfare (both as indicated in [10]). With respect to the representation of overall risk, it may be noted that all reported correlations of median expert scores with total RC scores were lower than the corresponding correlations with median RC scores (see Table 3). This may indicate that the procedure followed in the EFSA report [8,9] of leaving out some hazards for some housing systems reduced its suitability to derive overall welfare, as indicated in this study by expert opinion (but note that this was not an objective of the EFSA report, while it has been proposed from a semantic modelling perspective, [10]).

Veterinarians were the only group that showed a significant correlation with overall risk related to housing systems, namely -0.68 for median RC scores. Although this may suggest added value of consulting veterinarians in Risk Assessment as described in EFSA [8,9], it may also simply reflect their involvement in the report or a health-related underlying value in the EFSA [8,9] report (see [10]).

### Hazards

In this study, experts with a mixed background tended to give higher hazard scores than veterinarians, and ethologists gave intermediate scores close to the mixed-background experts. This could well indicate that welfare scientists may attach more importance to animal welfare than veterinarians do.

According to the experts, the least important hazards were insufficient human contact, separation from the dam, overfeeding and lack of maternal care. All other hazards had median scores of at least 6.0. The most important hazards (median scores > 6.5) were underfeeding, inadequate colostrum intake, poor education, insufficient space, insufficient roughage and iron deficiency, inadequate/inappropriate ventilation, poor floor conditions and no bedding (in that order). This list only partially confirms the analysis in EFSA ([8,9]; see also Figure 3), especially with respect to the importance of colostrum intake and inadequate ventilation. Insufficient light and mixing of calves were found to be much less important in the survey compared with the HC scores reported in EFSA [8,9]; e.g. mixing of calves was identified there as a main risk for calf welfare). As can be noted from the Boxplot shown in Figure 2, experts with a mixed background gave relatively high scores for these two hazards. Subsequent data exploration (not shown) indicated that Working Group members might have accounted for this difference, indicating that the discrepancy for these two hazards would be even larger if Working Group members who had written the report had been excluded from the analysis. This is in accordance with a previous suggestion [10], that the EFSA results may be diverging from current expert opinion. This is also true for several other hazards such as stockman education and to some extent (particularly for ethologists) access to a natural teat, which, conversely, seem to have been considered more important by the consulted experts than indicated by their HC scores reported in EFSA [8,9]. Furthermore, the median score of 7.0 for 'poor floor conditions' supports its ranking as 4^th ^most important hazards-class in Anonymous [11] and suggests a higher importance compared to the scores given in the EFSA report, where this hazard had been divided into 5 component hazards (see [10]). In addition, roughage was identified by the experts as an important hazard (median: 7.0), especially by experts with a mixed background (see Figure 2). This item had been added to the list, because it was considered to be either lacking from the EFSA report, or inadequately referred to by the hazard 'insufficiently balanced solid food' (HC = 3), again confirming our analysis in Bracke et al. [10]. The present study, however, did not confirm a similar hypothesis for the added hazard 'space to play' (median score: 6.0), which was rated as of average importance only (though it was still scored as more, but not significantly more, important than insufficient light and mixing of calves). A specific explanation for this finding cannot be provided, because experts did not specify the reasons for their scores (for feasibility reasons).

In this survey, underfeeding was the most important hazard. This may not appear to be surprising, because food has been identified as the 'gold standard' resource in consumer demand studies [24]; feed refusal is often a first and important sign of illness; and food is a necessary requirement for survival, growth, health and (re-)production. Given these scientific arguments it is surprising that, previously, underfeeding had not been identified as a main hazard by a group of 22 experts [11], and that it had received a Hazard Characterization (HC) score of only 4 on the 5 point scale in the EFSA (2006a, b) report. In the report, underfeeding was given the same HC score as, for example, high humidity, poor air quality (ammonia, dust) and continuous restocking (no all-in, all-out), but it received a lower HC score than, for example, inadequate ventilation, poor air quality (H_2_S), insufficient space, insufficient light, social isolation and mixing of calves from different sources. It would seem difficult, if not impossible, to justify these differences based on available scientific evidence. A possible explanation for the absence of underfeeding in Anonymous [11] can be found in the EFSA [8,9] report, where, in accordance with expectation, overall risk associated with underfeeding was low (even classified as 'negligible risk'), because whereas the effect (HC) was reasonably high, the occurrence probability, i.e. Exposure Assessment (EA) scores, were low (1 or 2 on a 5 point scale). In other words, in intensive calf rearing systems, aimed at maximized growth, underfeeding is rare. This corresponds to the procedure described in semantic modelling (SM) to exclude the above mentioned scientific evidence in the formal calculation of the weighting factor for underfeeding (which is equivalent to the HC score in Risk Assessment, see [10]), because it does not apply to the assessment domain where good farming practices were assumed. Technically, when the assessment domain only contains housing systems where animals are provided with sufficient food (as is normally the case in modern production systems), underfeeding is in fact much less important than at first suggested.

### Housing systems

The two 'control' systems added to the list of systems taken from EFSA [8,9] as part of our 'validation' effort [10], i.e. baby boxes and suckler beef calves at pasture, indeed obtained the lowest and highest predicted mean overall-welfare score, and they also differed significantly from all other systems. However, whereas the latter could be regarded as a true positive control, defining the upper range of calf welfare, the former system, baby boxes, cannot be regarded as a true negative control, as will be explained below.

Our finding that Working Group members did not significantly differ from reference authors indicates that the authors of the EFSA [8,9] report were 'in line' with the authors of their sources. Significantly higher scores given by Working Group members compared with veterinary exposure assessors and contacted experts were mainly due to higher scores for the 4 high-welfare systems (groups of dairy and sucker beef calves).

Veterinarians gave significantly higher scores for housing systems than either mixed-background experts or ethologists. As this was especially the case for the low-welfare (veal and feedlot) systems, the finding may correspond with their lower scores for hazard importance, confirming that veterinarians may have been less concerned by welfare problems in intensive systems for rearing calves than applied ethologists, whether or not they had a veterinary background. Other explanations, however, are also possible, e.g. that different definitions of animal welfare were used (despite the fact that welfare had been defined in the survey's instructions), perhaps involving a different weighting of welfare aspects (e.g. physical versus mental health; physiological versus behavioural needs). Such differences would be expected between experts with different backgrounds, given, for example, the fact that many years of dispute has not yet resulted in a commonly accepted definition of animal welfare among ethologists [11,25,10].

Veterinarians gave higher median scores to each of the 5 veal systems, especially for baby boxes, compared with ethologists and experts with a mixed background (see Figure 1). Median veterinary scores did not drop below 5.0 for any housing system. Their lowest median scores, given for feedlots, white veal in small groups, and baby boxes, were 5.0, 5.3 and 6.0 respectively. This implied that baby boxes were not a negative control system for veterinarians, because they gave lower (though not significantly lower) scores to the two other systems. By contrast, animal welfare experts, i.e. ethologists and experts with a veterinary background working in applied ethology, were much more negative about these three systems (medians between 0.0 and 4.0), and ethologists gave scores below 6 also to other veal systems, to hutches and to small groups of dairy calves (see Figure 1). This apparent difference may be related to differences in professional experience and affinity to health and production in the sector (despite what was claimed about the veterinarians' independence in the EFSA report, see [10]). This hypothesis could, for example, explain why veterinarians gave relatively high scores for calves kept in baby boxes (and hutches), as individual housing promotes hygiene. It could, perhaps, also explain why they identified feedlots, an American system which is not prevalent in Europe, as the worst system. Finally, it could explain that veterinarians showed lower median scores for access to a natural teat and for castration/dehorning (see Figures 2 and 3), because natural teat sucking is a typical behavioural requirement and castration/dehorning is very much part of routine veterinary practice.

## Conclusion

This paper reported a 'validation' study of the EFSA report, comparing its scores for hazard characterization (HC) and Risk Characterization (RC) with semantic-modelling type scores elicited from a limited number of experts about a selected number of welfare hazards and housing systems for calves according to recommendations formulated in the underlying paper [10]. Experts included ethologists and veterinarians involved in the publication of the EFSA report, as well as authors of publications cited in that report.

Differences in welfare score were found between housing systems, between hazards, between experts with different types of involvement in the EFSA report [8,9], and between experts with different backgrounds.

The poorest welfare systems (with median scores < 5.5) were: white veal housed individually in baby boxes (first 6–8 weeks), bucket fed (i.e. not suckling); white veal in small groups, bucket fed (i.e. not suckling); feed lots (high density groups within outside pen); and white veal in larger groups, with automatic feeding (i.e. not suckling). The best system was: suckler beef calves kept with cows in a herd at pasture.

The most important hazards (with median scores > 6.5) were: underfeeding; inadequate colostrum intake; poorly educated stockperson; insufficient floor space allowance; insufficient/inadequate roughage; iron deficiency; inadequate/inappropriate ventilation; poor floor conditions; and no bedding.

This study provided only limited support of the scores reported in EFSA [8,9] and emphasized a number of suggestions identified in Bracke et al. [10] to improve the EFSA [8,9] Risk Assessment from the perspective of semantic modelling. Hazards and housing systems should be specified in more detail in relation to the available scientific information. Risk Characterization (RC) scores could be linked to overall welfare scores for housing systems, which are inherently lacking in Risk Assessment. Measures of uncertainty should be included, such as those based on the level of concordance among the experts or based on the variation in scores. Rules could be specified for including and leaving out items (hazards or housing systems) from a list, e.g. by including positive 'hazards', i.e. benefits for welfare such as play and roughage for calves, and by including items that define the end points of the scale, such as illustrated by the positive and negative 'controls' in the list of housing systems. Finally, transparency about underlying values is called for, as this study confirmed some widely perceived differences in professional backgrounds of experts and the impact of these differences on their perception of animal welfare.

These points illustrate that, for further development, Risk Assessment applied to animal welfare can benefit from semantic modelling principles, and that both can benefit from improved definitions of important concepts such as welfare, its components, hazards and risks.

## Competing interests

The authors declare that they have no competing interests.

## Authors' contributions

MBMB did most of the data collection and writing. BE en WGB were involved as statisticians. SEA and BA provided most helpful comments and guidance in setting up the work and in getting it published. All authors read and approved the final manuscript.
